# Cr(III) Ion-Imprinted Hydrogel Membrane for Chromium Speciation Analysis in Water Samples

**DOI:** 10.3390/gels8110757

**Published:** 2022-11-21

**Authors:** Ivanka Dakova, Penka Vasileva, Irina Karadjova

**Affiliations:** Faculty of Chemistry and Pharmacy, University of Sofia “St. Kliment Ohridski”, 1, J. Bourchier Blvd., 1164 Sofia, Bulgaria

**Keywords:** ion-imprinted hydrogel membrane, Cr(III), sodium alginate, polyvinyl alcohol, gold nanoparticles, chromium speciation, surface waters

## Abstract

Novel Cr(III)-imprinted poly(vinyl alcohol)/sodium alginate/AuNPs hydrogel membranes (Cr(III)-IIMs) were obtained and characterized and further applied as a sorbent for chromium speciation in waters. Cr(III)-IIMs were prepared via solution blending method using blends of poly(vinyl alcohol) and sodium alginate as film-forming materials, poly(ethylene glycol) as a porogen agent, sodium alginate stabilized gold nanoparticles (SA-AuNPs) as a crosslinking and mechanically stabilizing component, and Cr(III) ions as a template species. The physicochemical characteristics of pre-synthesized AuNPs and obtained hydrogel membranes Cr(III)-IIM were studied by UV-vis and FTIR spectroscopy, TEM and SEM observations, N_2_ adsorption–desorption measurements, and XRD analysis. The mechanism of the adsorption process toward Cr(III) was best described by pseudo-first-order kinetic and Langmuir models. Experiments performed showed that quantitative retention of Cr(III) is attained in 20 h at pH 6 and temperature 40 °C. Under the same conditions, the adsorption of Cr(VI) is below 5%. A simple and sensitive analytical procedure was developed for the speciation of Cr in an aquatic environment using dispersive solid phase extraction of Cr(III) by Cr(III)-IIM prior to selective Cr(VI) measurement by ETAAS in the supernatants. The detection limits and reproducibility achieved for the Cr speciation analysis fulfill the requirements for their monitoring in waters under the demand of the Water Framework Directive.

## 1. Introduction

The importance of selective and sensitive determination of the two most common chemical forms of chromium, Cr (III) and Cr (VI), demanded by their very different toxic effects, is still an analytical problem. In contrast to the relatively non-toxicity of Cr(III), Cr(VI) is highly toxic to most living organisms, causing strong adverse effects and diseases [1]. Chromium exists mostly as Cr(III) in the aquatic environment; toxic Cr(VI) is also present, however, at much lower concentrations as a result of its ongoing industrial application. That is why developed speciation methods should allow direct, selective determination of low levels of toxic Cr(VI) in order to ensure reliable speciation results. Moreover, considering the high oxidizing power and chemical activity of Cr(VI), the proposed method should preserve the original concentrations of Cr(III) and Cr(VI) during the sample transportation to the analytical laboratory, meaning that the separation step should be performed during sampling. From such a point of view, the creation of conditions for the separation of the two chemical forms immediately after sample collection and during its transportation is both preferable and encourages the preparation of innovative materials for non-chromatographic, selective determination of toxic Cr(VI). Very recently, advanced techniques for the selective removal of Cr(VI) from aqueous samples have been presented in a review article [2]. Modern analytical strategies and efficient nanosized sorbents used for chromium speciation in various matrices have been summarized and critically discussed in several review articles [3,4,5,6,7,8].

The application of ion-imprinted polymers (IIPs) as sorbents for elemental speciation analysis attracts extensive research interest due to their advantages, such as selectivity, stability, ease of preparation, low cost, and reusability [9]. Several Cr(III)-IIPs have been studied and characterized as effective sorbents in non-chromatographic speciation analysis of chromium [10,11,12,13,14,15]. These polymer sorbents have been synthesized as micro- or nanoparticles so that the proposed analytical procedures could not avoid the filtration/centrifugation steps. This drawback might be overcome by using membranes instead of particles as sorbents for solid phase extraction (SPE). It is known that hydrogel membranes can be successfully used to adsorb pollutants from water samples [16]. Hydrogel membranes are crosslinked three-dimensional (3D) networks composed of hydrophilic polymers (natural or synthetic). Their selectivity might be additionally improved by the introduction of the ion template species resulting in the high recognition ability of ion imprinted polymers [17,18]. Studies on the synthesis of IIMs and their application for selective adsorption of Cr(III) [19,20] or Cr(VI) [21,22] ions, mostly from water samples, have been reported, but no studies are known about the green synthesis of Cr(III)-IIMs and their application for chromium speciation analysis.

One of the strategies for the green synthesis of hydrogel membranes is based on the use of renewable or natural materials in the membrane formation process. Sodium alginate (SA) is a natural, non-toxic, biocompatible, and biodegradable anionic polysaccharide composed of 1,4-linked β-D-mannuronic acid and 1,4-linked α-L-guluronic acid residues, containing carboxyl and hydroxyl groups [23]. It is well known as an environmentally friendly polymer for membrane preparation. Poly(vinyl alcohol) (PVA) is characterized by properties such as non-toxicity, biocompatibility, high hydrophilicity, film-forming ability, and chemical and mechanical resistance [23]. Blending SA with PVA results in polymeric materials that possess the desired properties, such as improved physical characteristics and film-forming abilities. Since traditionally used crosslinking agents for SA and PVA, such as epichlorohydrin and glutaric dialdehyde, are toxic [24,25], it is recommended to use inorganic crosslinking agents [26]. For example, gold nanoparticles (AuNPs), known to be non-toxic [27], could be used in this case. A significant additional advantage of AuNPs as cross-linkers is their capacity to form multiple bonds (so-called multivalency) within the gel networks [28].

In the present work, Cr(III)-IIMs are synthesized using sodium alginate and poly(vinyl alcohol) as film-forming materials, poly(ethylene glycol) (PEG) as a porogen agent, SA-coated gold nanoparticles (SA-AuNPs) as a crosslinking and mechanically stabilizing component, and Cr(III) ions as template species. The literature survey showed that the preparation of Cr(III)-imprinted PVA/SA/AuNPs hydrogel membrane has not yet been published. The physicochemical characteristics of pre-synthesized SA-AuNPs and hydrogel Cr(III)-IIM were studied by UV-vis and FTIR spectroscopy, TEM and SEM observations, and XRD analysis. Cr(III) imprinting is confirmed by FTIR spectroscopy and sorption characteristics by N_2_ adsorption–desorption measurements. Experiments performed showed that the mechanism of sorption of Cr(III) was best described by pseudo-first-order kinetic and Langmuir models. A novel analytical procedure for solid phase extraction, which combines the selectivity of ion imprinting with the practicality of hydrogel membrane application, is developed for direct selective determination of toxic Cr(VI) in water samples. The procedure proposed might be performed in one reaction vessel, avoiding desorption steps and any operations leading to contamination or loss of analyte. Moreover, the whole procedure for Cr(VI) quantification could be performed during sampling on a membrane previously transferred in a polypropylene vessel and brought to the sampling site.

## 2. Results and Discussion

### 2.1. Cr(III) Ion-Imprinted Hydrogel Membrane Synthesis

**Figure 1:**
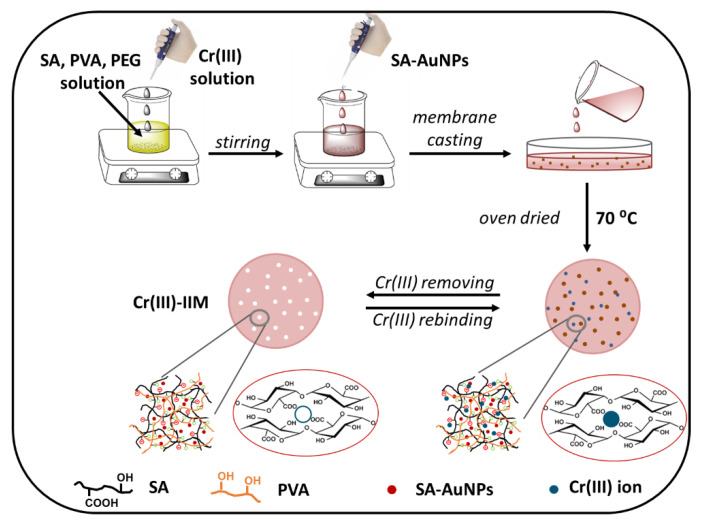
Schematic representation of the hydrogel Cr(III)-IIMs preparation.

Cr(III)-IIMs were synthesized by an approach based on “crosslinking of linear chain polymers” [29]. In the preparation process of Cr(III)-IIM adsorbent, two kinds of polymer materials, SA and PVA, were used as the functional hydrogel matrix. Preparation of Cr(III)–IIM included several steps, shown schematically in Figure 1. Initially, the solution of SA was blended with PVA, and then a solution of Cr(III) ions (template) was added. As a result, the carboxylate ions of SA formed complexes with Cr(III) cations, while the hydroxyl groups of SA could form coordinate bonds with Cr(III), confirmed by FTIR spectra. Based on these two kinds of chemical bonds, many stable structures like “egg box” were formed by SA chains [23]. In the next step, PVA chains were physically crosslinked with SA-AuNPs due to the coordination interaction between sodium alginate-capped AuNPs and hydroxyl groups of PVA [30]. Then the hydrogel matrix dispersion prepared was cast on the bottom of glass beakers and allowed the solvent to evaporate and dry until the formation of the membranes. The obtained self-standing hydrogel membrane can be considered a novel double crosslinking interpenetrating polymer network [31]. In the final step, Cr(III) ions were removed from the membrane prepared, resulting in the formation of a cavity with geometry and functional groups oriented specifically to the complex formation with template specie.

### 2.2. Characterization of SA-AuNPs and Cr(III)-IIM

The optical, morphological and structural properties of SA-AuNPs before and after their incorporation in the hydrogel polymer matrix of membranes are studied and compared.

**Figure 2:**
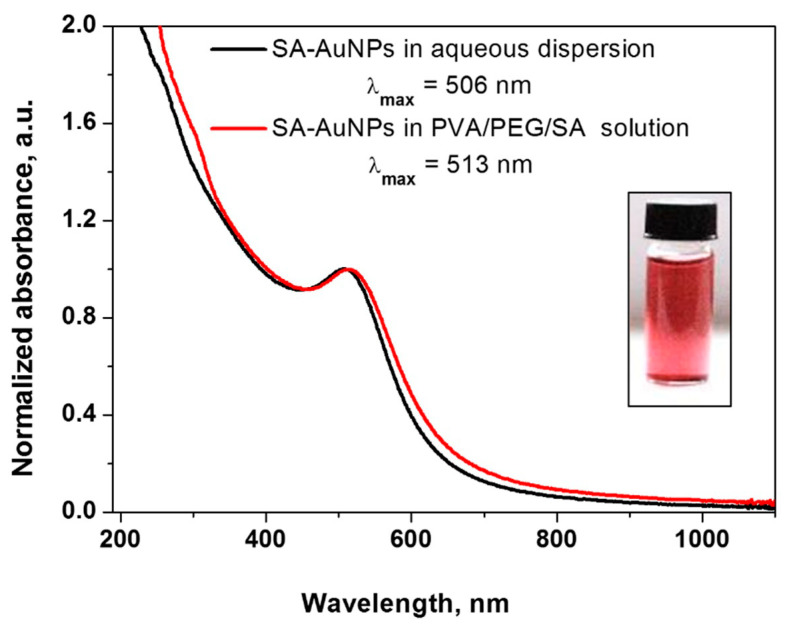
UV-vis absorption spectra of SA-AuNPs in: aqueous dispersion (red line) and PVA/PEG/SA hydrogel matrix solution (black line); inset: optical photo of SA-AuNPs aqueous dispersion.

The UV-vis absorption spectrum of SA-AuNPs, recorded right after their preparation by sodium borohydride reduction of AuCl_4_^−^, is shown in Figure 2. The pink-red SA- AuNPs dispersion shows a surface plasmon resonance (SPR) band at 508 nm, and no aggregation was observed for at least up to six months. The narrow absorption band suggests the preparation of small gold nanoparticles with a narrow size distribution, as further confirmed by the TEM and XRD analysis. Figure 2 also displays the effect of SA-AuNPs incorporation in PVA/PEG/SA hydrogel polymer matrix solution before casting the membranes on the optical properties of gold nanoparticles. A red shift of the absorption band of gold nanoparticles (from 508 nm to 515 nm) was observed after the incorporation of AuNPs in the hydrogel polymer matrix, probably due to the partial sintering. No aggregation was observed, as further confirmed by TEM observation.

**Figure 3:**
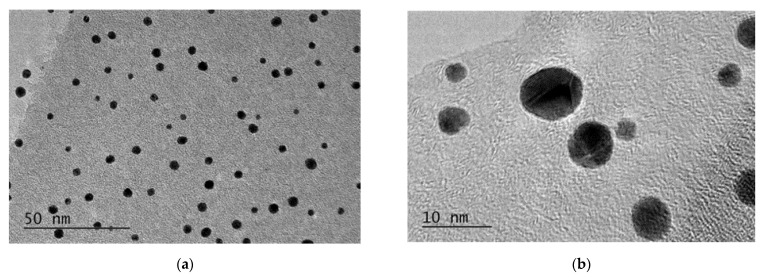
(**a**) TEM and (**b**) HRTEM micrographs of SA-AuNPs.

Figure 3 shows TEM images at different magnifications of gold nanoparticles prepared by chemical reduction of AuCl_4_^−^ and stabilized by sodium alginate.

Most gold nanoparticles have a nearly spherical morphology, while a small part of them is polyhedral. It can be clearly seen that the nanoparticles in the aqueous dispersion are well separated from each other due to the protection by SA and have a small particle size—the average particle diameter is 4.9 ± 0.6 nm. An insignificant number of very small gold nanoparticles are also seen in TEM micrographs, which confirms the effective stabilization of nanoparticles with SA in aqueous dispersion.

**Figure 4:**
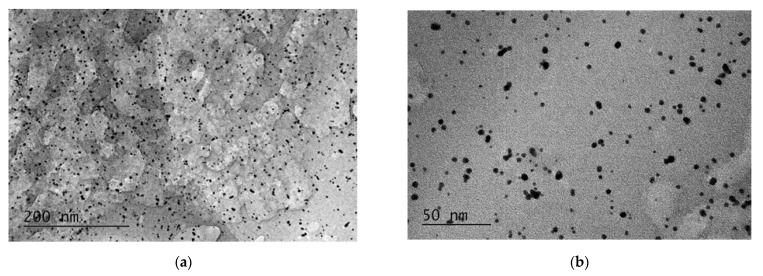
TEM micrographs at different magnifications (**a**,**b**) of Cr(III)-IIM.

TEM micrographs of hydrogel Cr(III)-IIM (Figure 4) display uniformly distributed gold nanoparticles with efficient stabilization by SA throughout the entire Cr(III)-imprinted PVA/SA hydrogel network. Local congregations of gold nanoparticles in the Cr(III)-IIM are observed in the TEM micrograph at higher magnification, probably due to the role of Cr(III) ions as a linker between nanoparticles, a natural result of which is a reduction of the distances between them in the ion-imprinted membrane. This observation is in excellent agreement with the previously commented red shift of the SPR band of AuNPs after their incorporation into the PVA/PEG/SA polymer hydrogel matrix.

An energy dispersive X-ray (EDX) elemental analysis was conducted for further investigation of the Cr(III)-IIM surface characteristics. The results are shown in Figure S1a,b. The EDX mapping confirmed the homogeneous dispersion of both Cr and Au elements in the polymer hydrogel matrix of the membrane. From the EDX spectrum and the inset table of Figure S1b, giving the elemental composition of Cr(III)-IIM, the presence of Cr and gold nanoparticles is confirmed.

**Figure 5:**
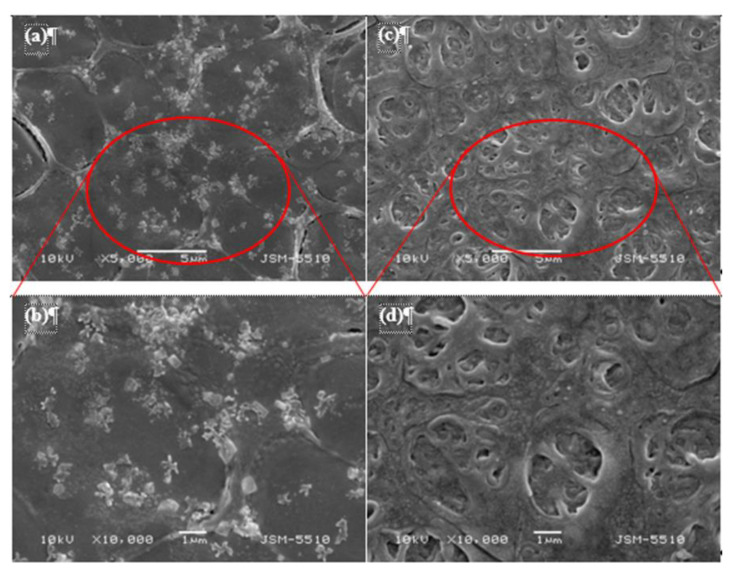
SEM images at different magnifications of (**a**,**b**) NIIM and (**c**,**d**) Cr(III)-IIM.

The surface morphology characteristics of a non-imprinted membrane (NIIM) and Cr(III)-IIM were compared using SEM. As shown in Figure 5, the Cr(III)-IIM and NIIM display considerable differences in surface morphology. The SEM images of NIIM (Figure 5a,b) represent non-uniformity and some conglomeration of SA-AuNPs on the membrane surface. In contrast, the SEM images at different magnifications of Cr(III)-IIM (Figure 5c,d) clearly indicate a more uniform distribution of SA-AuNPs. Surface pores of Cr(III)-IIM can be distinguished with average sizes around 0.2–0.3 μm, while for NIIM, there are no pores on the membrane surface. The formation of a double crosslinking interpenetrating polymer network in Cr(III)-IIM can help generate a regularly distributed surface morphology, which does not exist in NIIM since Cr(III) ions are absent.

FTIR spectroscopy was used to elucidate the structure of hydrogel Cr(III)-IIM (see Figure S2). The chelate complex formation between alginic acid and metal ions is thoroughly studied, and the structure of complexes formed is confirmed by FTIR spectroscopy in the published literature [19,32]. As expected, a comparison of FTIR spectra of NIIM and Cr(III)-IIM shows that bands of the asymmetric (ν_as_) and symmetric (ν_s_) stretching vibrations of alginic acid −OCO− group are shifted from 1654 cm^−1^ and 1419 cm^−1^ for NIIM to lower frequencies of 1601 cm^−1^ and 1409 cm^−1^ for Cr(III)-IIM, respectively. These results mean that carboxylic functional groups take part in chelate formation. The shift of the broad ν_OH_ band at around 3400 cm^−1^ to lower frequencies indicates that the OH groups are also involved in the chelation. FTIR spectra proved the coordination process between Cr(III) and alginic acid confirming the successful imprinting of Cr(III) in hydrogel Cr(III)-IIM. A schematic presentation of the interactions between SA and Cr(III) ions is shown in Figure S3.

Nitrogen adsorption–desorption isotherm studies performed for hydrogel Cr(III)-IIM membrane indicated that the specific surface area is 5 m^2^/g with a total pore volume of 0.04 cm^3^/g. Similar results have already been reported for hydrogel membranes based on SA/PVA blend and different inorganic constituents [33,34].

The XRD pattern of the PVA/PEG/SA polymer membrane shows a significant semicrystalline peak at 2θ value of 19.65°, which is connected to the PVA structure, generated from strong intra- and intermolecular hydrogen bonding [35,36] (see Figure S4a). In cases of NIIM and Cr(III)-IIM, this semicrystalline peak appears at the same 2θ value along with other broad diffraction peaks of low intensity centered at 2θ values of 38.8°, 44.4°, 64.7°, 77.5°, which can be indexed to the (111), (200), (220), and (311) crystal planes corresponding to the face-centered crystal (fcc) structure of gold [37] (see Figure S4b).

### 2.3. Adsorption Behavior of Cr-IIM toward Cr(III) and Cr(VI)—Optimization Studies

**Figure 6:**
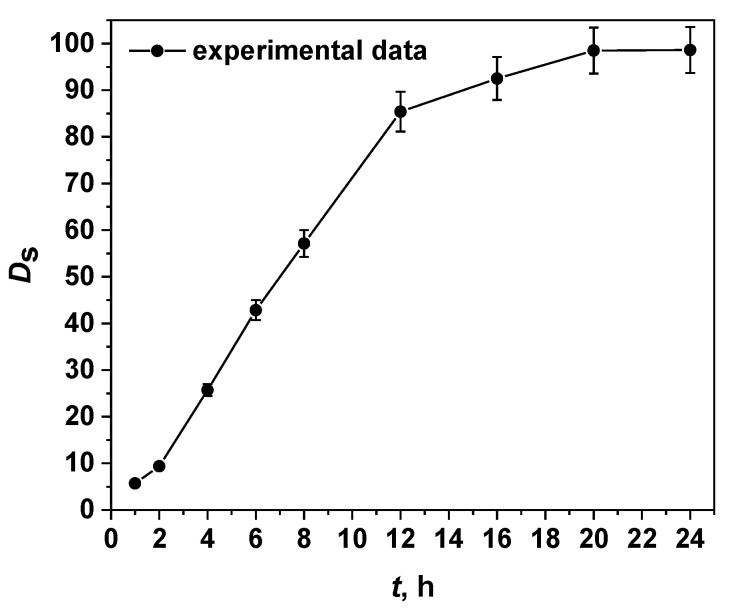
Effect of contact time on the degree of sorption *D*_s_ of Cr(III) onto Cr(III)-IIM at initial concentration 5 mg/L, pH 6, temperature 40 °C, and adsorbent dose (one membrane) 0.140 g.

In order to evaluate the suitability of hydrogel Cr(III)-IIM as a sorbent for the selective separation of Cr(III) ions, chemical conditions for quantitative retention of Cr(III) were optimized. Taking into account the kinetic inertness of Cr(H_2_O)_6_^3+^ complexes, three important parameters were optimized—pH, temperature, and time for adsorption. As a first step, the progress of Cr(III) retention on the Cr(III)-IIM at different times was studied. Experimental data for the degree of Cr(III) sorption, *D*_s_, were obtained at initial concentration 5 mg/L, pH 6, and temperature 40 °C. The kinetic adsorption curve is shown in Figure 6, where the duration of the sorption process varied from 1 to 24 h. It can be seen that as the contact time increases, the degree of sorption *D*_s_ also increases. According to this curve, quantitative sorption > 95% for Cr(III) in the Cr(III)-IIM adsorption system was achieved within 20 h. The retention time considered optimal was set to 20 h. A similar relatively slow process (equilibrium time of 18 h) has already been reported for quantitative Pd(II) sorption using palladium imprinted membrane based on a chitosan matrix with azo-derivative ligand [38]. It is reasonable to assume that such a slow reaching of the adsorption equilibrium is due to the large diffusion barrier in the thin ion-imprinted membrane. The greater diffusion resistance leads both to the difficult entry of Cr(III) ions into the membrane cavities and to their limited association with the recognition centers.

The acidity of the solution is an important parameter determining the effectiveness of the SPE procedure because the pH value affects both the binding sites on the surface of the sorbent and the metal chemistry in aqueous solutions. In order to preserve the original concentrations of Cr(III) and Cr(VI) during the Cr(III) sorption onto Cr(III)-imprinted PVA/SA/AuNPs membrane, it is very important to take into account the possibility of reduction of Cr (VI) by the carboxyl groups of SA—a process that also depends on pH. The influence of pH on the reduction of Cr(VI) to Cr(III) with alginic acid is well established—at pH 1–3, alginic acid slowly reduces Cr(VI); at pH 6.0, the redox reaction of Cr(VI) with alginic acid proceeds very slowly, with negligible reduction of Cr(VI) [39].

**Figure 7:**
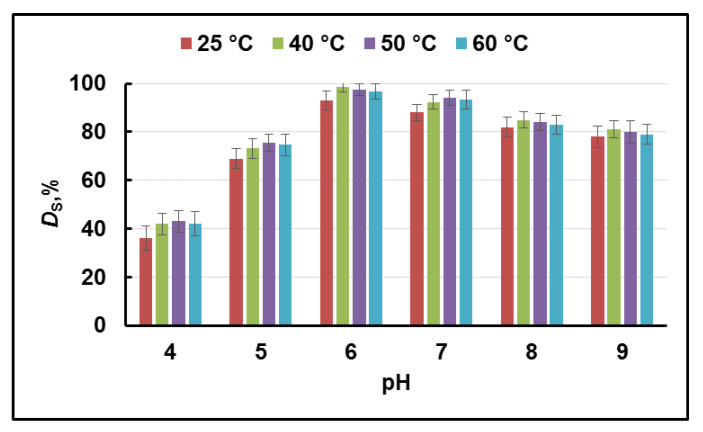
Dependence of the degree of sorption (*D*_S_, %) of Cr(III) ions onto Cr(III)-IIM on pH and temperature.

The effect of pH (in the range 4–9) and temperature (25, 40, 50, and 60 °C) on the degree of Cr(III) sorption onto Cr(III)-IIM is illustrated in Figure 7. The hydrogel membranes prepared contain carboxylic groups (in SA) and hydroxylic groups (in SA and PVA) in the polymer matrix, suggesting that at lower pH (pH < pKa = 3.6 for alginic acid), the functional groups are protonated, and in this way, the Cr(III) adsorption onto Cr(III)-IIM is restricted. Hence, the values of *D*_s_ are very small (these results are not presented in Figure 7). It is seen from Figure 7 that the degree of Cr(III) sorption is enhanced with an increasing pH of 6 for all studied temperatures. At pH values in the range 4–6, the fraction of deprotonated carboxyl groups in SA grows and (–COO^−^) becomes available for binding and adsorption of Cr(III) cations. In addition, the positively charged Cr^3+^ and CrOH^2+^ ions (species existing at pH < 6 [40]) can be bound to the negatively charged groups of the membrane by electrostatic attraction, leading to an increased degree of sorption. However, at pH values higher than 6, a decrease in the degree of Cr(III) sorption is noticed (Figure 7), which may be attributed to the precipitation of the metal ions as Cr(OH)_3_ [40]. Furthermore, the temperature dependence of the degree of Cr(III) sorption on Cr(III)-IIM is clearly visible from the results in Figure 7.

**Figure 8:**
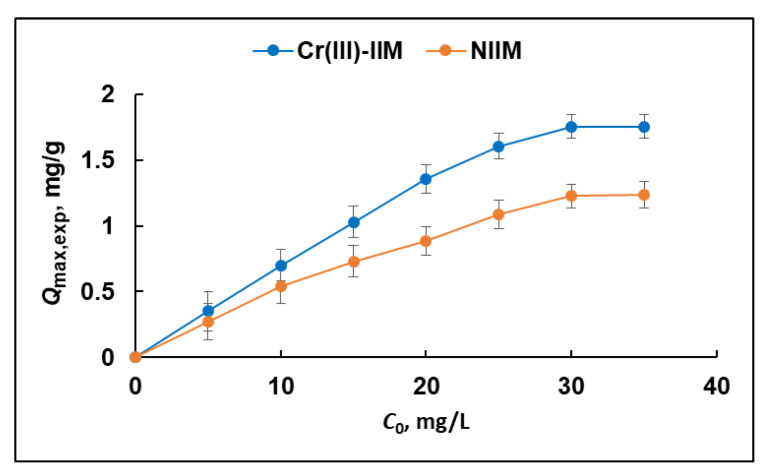
Effect of the initial concentration of Cr(III) on the adsorption capacity of Cr(III)-IIM and NIIM (pH 6; contact time 20 h; temperature 40 °C).

Quantitative Cr(III) sorption (*D*_s_ > 95%) is achieved at temperatures in the range of 40–50 °C, while the degree of Cr(III) sorption is lower at temperatures outside this range (91.0% and 93.7% at temperatures of 25 °C and 60 °C, respectively). These results can be explained by the kinetic stability of the Cr(H_2_O)_6_^3+^ complex, for which ligand exchange in the inner coordination sphere requires elevated temperatures. Finally, quantitative retention of Cr(III) on the Cr(III)-IIM was achieved at optimal pH 6 for 20 h at a temperature of 40 °C. Under the established optimal conditions for quantitative sorption of Cr(III), the degree of Cr(VI) sorption is found to be less than 5%. These results unambiguously confirm that the hydrogel Cr(III)-IIM can be used for the quantitative separation of Cr species in order to perform successful speciation analysis. Results from parallel adsorption experiments carried out with NIIM membrane showed similar sorption behavior (not presented in Figure 7) toward Cr(III), however, with about a 30% lower value of *D*_s_. Under defined optimal conditions, the sorption capacity of the Cr(III)-IIM and NIIM were evaluated after saturation of the membranes with Cr(III) ions. The effect of the initial concentration of Cr(III) ions (5–35 mg/L) on the sorption capacity of Cr(III)-IIM and NIIM is displayed in Figure 8.

Adsorption isotherms (Figure 8) clearly show that the amount of adsorbed Cr(III) per unit mass of the membrane increases with growing Cr(III) concentration and reaches a plateau determining the maximum adsorption capacity (*Q*_max,exp_)—1.75 mg/g for Cr(III)-IIM and 1.23 mg/g for NIIM. As expected, the adsorption capacity of Cr(III)-IIM exceeds the NIIM’s capacity, indicating that the binding sites created after the removal of template ions ensure higher affinity of the hydrogel Cr(III)-IIM toward Cr(III) in this way proving the advantages of ion-imprinting approach for the preparation of sorbent materials with higher adsorption capacity.

### 2.4. Elution Studies

The elution step should ensure quantitative desorption of sorbed Cr(III) in this way, ensuring further use of synthesized Cr(III)-IIM. Eluent solutions containing HCl or NH_4_-EDTA were tested for Cr quantitative extraction from loaded Cr(III)-IIMs. The results obtained are presented in Table 1. It can be concluded that hydrochloric acid at any concentration level is not suitable for the elution of Cr(III)—the elution is not quantitative, and AuNPs in the membranes are dissolved at the higher acid concentration (1 mol/L). The most suitable eluent is NH_4_-EDTA solution (0.1 mol/L), which provides complete elution of Cr(III) (>99%) from the membranes, and at the same time, the membrane composition and stability are unaffected. The effect of desorption agent volume was also studied (Table 1). A 10 mL NH_4_-EDTA solution was found to be the optimum volume to provide quantitative Cr(III) elution from the membranes. The kinetics of the Cr(III) desorption process studied according to the procedure described in Section 4.5 for 1–5 h showed that quantitative desorption was reached for 2 h. Optimal conditions defined for quantitative elution of Cr(III) include 10 mL 0.1 mol/L NH_4_-EDTA for 2 h desorption time.

### 2.5. Investigations on the Mechanism of Cr(III) Adsorption onto Cr(III)-IIM

#### 2.5.1. Adsorption Isotherm Models

The adsorption data for Cr(III)-IIM and NIIM as a function of the initial Cr(III) concentrations were analyzed using the Freundlich and Langmuir adsorption isotherm models. The applicability of the isotherm models was evaluated by comparing the calculated values for the *R*^2^ coefficient.

The Freundlich isotherm model can be applied in the case of multilayer adsorption of the adsorbate on a heterogeneous surface [41]. Equation (1) presents the Freundlich isotherm in the linear form:

(1) $$ln\;Q_{e}=ln\;k_{F}+n^{-1}.ln\;C_{e}$$

where *C*_e_ (mg/L) and *Q*_e_ (mg/g) are Cr(III) equilibrium concentration in the solution and equilibrium capacity of the membranes, respectively; *k*_F_ is the Freundlich isotherm constant; *n* is the adsorption intensity. The value of *n* gives information about the adsorbent–adsorbate interaction. The adsorption process is favorable, when 0 < 1/*n* < 1; unfavorable—1/*n* > 1; and irreversible—1/*n* = 1 [42].

The Langmuir isotherm model describes a sorption process occurring in a surface monolayer of homogeneous sites [41]. The linear form of Langmuir isotherm is presented by (Equation (2)):

(2) $$\frac{C_{e}}{Q_{e}}=\frac{C_{e}}{Q_{\max}}+\frac{1}{b.Q_{\max}}$$

where *Q*_max_ (mg/g) is the calculated maximum adsorption capacity, *b* (L/mg) is the Langmuir constant.

To predict the favorability of a given adsorption system, it is recommended to use the dimensionless factor *R*_L_ (Equation (3)). The isotherm is irreversible, favorable, linear, or unfavorable if *R*_L_ = 0, 0 < *R*_L_ < 1, *R*_L_ = 1, or *R*_L_ > 1, respectively [41].

(3) $$R_{L}=\frac{1\;}{1+b.C_{0}}$$

The final calculation results of Langmuir and Freundlich isotherm parameters are exhibited in Table 2, and the graphical visualization is in Figure S5.

From Table 2, it can be concluded that the values of coefficient of determination *R*^2^ obtained for the Langmuir model (0.9997 and 0.9993 for Cr(III)-IIM and NIIM, respectively) are higher than values obtained when using Freundlich isotherm (0.8956 and 0.9592 for Cr(III)-IIM and NIIM, respectively). The calculated values of adsorption capacity *Q*_max,calc_ are in good agreement with experimentally obtained values (Table 2). These results confirm the correctness of the assumption that the adsorption process occurs in a surface monolayer of homogeneous sites.

The calculated values of Langmuir dimensionless factor *R*_L_ are in the range 0 < *R*_L_ < 1 (Table 2), indicating that the adsorption of Cr(III) ions onto Cr(III)-IIM and NIIM is favorable. This conclusion for favorable adsorption is also confirmed by the values of the Freundlich coefficient *n* related to the adsorption intensity that satisfies the condition 0 < 1/*n* < 1 (1/*n* is 0.28 and 0.46 for Cr(III)-IIM and NIIM, respectively).

#### 2.5.2. Modeling of Cr(III) Sorption Kinetics

In order to understand the behavior of Cr(III) ions adsorbed by the novel hydrogel Cr(III)-IIM and to determine the controlling mechanism of the adsorption process, several kinetic models, which contain two undetermined parameters, have been used to fit the experimental data [43]:

(4) $$\mathrm{pseudo}\text{-}\mathrm{first}\text{-}\mathrm{order}\;\mathrm{model}:\;q_{t}=q_{e\;}\left(1-e^{-k_{1}.t}\right)$$

where *q*_t_ and *q*_e_ (mg/g) are the adsorbed amounts at different times *t* (h) and at an equilibrium, respectively, and *k*_1_ (1/h) is the rate constant. The pseudo-first-order kinetic model better describes an adsorption process controlled by diffusion and is mainly used to simulate a simple single reaction.

(5) $$\mathrm{pseudo}\text{-}\mathrm{second}\text{-}\mathrm{order}\;\mathrm{model}:\;q_{t}=k_{2\;}.q_{e}^{2}\frac{t}{1+k_{2}.q_{e}.t}$$

where *k*_2_ (g/(mg∙h) is the rate constant. The pseudo-second-order model assumes that the chemisorption is a rate-limiting step.

(6) $$\mathrm{Elovich}\;\mathrm{equation}:\;q_{t}=\frac{1}{\beta }\ln_{\;}\left(\alpha .\beta \right)+\frac{1}{\beta }\ln t\;$$

where *α* (mg/(g∙h)) is the initial rate of the adsorption process and *β* (g/mg) is the desorption constant of this process related to the extent of surface coverage and activation energy of chemisorption. Elovich equation is useful in describing the chemical sorption on highly heterogeneous surfaces [44].

In order to find other important correlations of experimental kinetic data in this study, the Weber and Morris equation was tested for evaluation of adsorption kinetics of Cr(III) ions onto Cr(III)-IIM:

(7) $$\mathrm{intra}\text{-}\mathrm{particle}\;\mathrm{diffusion}\;\mathrm{model}:\;q_{t}=k_{i\;}.\;t^{0.5}+C_{i}$$

where *k*_i_ (mg/(g∙h^0.5^)) is the equilibrium rate constant of intra-particle diffusion, and *C*_i_ (mg/g) is the intercept associated with the thickness of the boundary layer. The intra-particle diffusion model describes the kinetics of the diffusion process inside a particle; it is not suitable for describing the kinetics of the diffusion process on the surface of a particle [45].

Kinetic parameters of pseudo-first-order, pseudo-second-order, and Elovich kinetic models estimated by regression analysis are summarized in Table 3, and the fitted curves are plotted in Figure S6a–c. To choose the superior model, both coefficient of determination (*R*^2^) and the equilibrium adsorption capacity predicted by the model (*q*_e_,_calc_) should be considered [46].

The low value of the determination coefficient (0.9331, Table 3) shows that the Elovich model is unsuitable to represent the adsorption of Cr(III) ions onto the Cr(III)-IIM and also indicates that the adsorption process is not controlled by chemisorption [13]. Curve fitting results (Table 3) implied that the pseudo-first order kinetic model (*R*^2^ = 0.9694) is more suitable to describe the adsorption behavior than the pseudo-second order model (*R*^2^ = 0.9626), and the values of *q*_e,exp_ and *q*_e,calc_ are closer to each other under pseudo-first-order kinetic model than that of pseudo-second-order model, indicating that the adsorption is mainly controlled by diffusion.

The rate constant of intra-particle diffusion *k*_i_ could be obtained from the slope of the plot presented in Figure S6d. It is seen that the plot does not pass through the origin and is nonlinear. It can be concluded that the adsorption of Cr(III) ions onto Cr(III)-IIM is a complex process [47]. Two straight lines simulating the experimental results and the values of kinetic parameters are presented in Table 3. The slope of the line for the first region (responsible for external diffusion; *k*_i,1_ = 0.10342) is higher than the slope of the line for the second region (corresponding to intra-particle diffusion; *k*_i,2_ = 0.03459), which confirms the conclusion that the active sorption sites for Cr(III) ions are distributed onto the outer sorbent surface and penetration into the inside of the membrane is insignificant [48]. A negative *C_i_* value in Equation (7) (see Table 3, Region 1) could be explained by the combined effects of surface reaction control and film diffusion processes [49].

### 2.6. Analytical Applications

The experimental results obtained showed that an analytical procedure for Cr speciation might be developed based on the sorption of Cr(III) on the hydrogel membrane and selective determination of Cr(VI) in the supernatant (see Section 4.7). Model experiments were performed with various waters such as river, sea, and mineral water aiming to assess the selective recovery of Cr(VI) independent of the water matrix. As a first step, interference studies according to the procedure described in Section 4.6 were performed in order to confirm that even in the presence of different levels of matrix cations and anions, quantitative separation of both Cr(III) and Cr(VI) is still achieved (see Table S1). Results obtained undoubtedly showed that independently of the sample matrix degree of sorption of Cr(III) is in the range between 95–98%, and for Cr(VI), in all cases degree of sorption is below 5%. As a next step, the separation of both species was studied at different ratios more relevant to the environmental conditions, e.g., relatively low concentrations of Cr(VI) in the presence of high amounts of Cr(III) and for different types of surface waters, using added/found method. River water, groundwater, and seawater, filtered through a cellulose membrane filter (0.45 µm), were spiked with different concentration ratios of Cr(VI) to Cr(III) and passed through the procedure described in Section 4.7. The results obtained are presented in Table 4.

Evidently, for all studied ratios and for all types of waters, recoveries for toxic Cr(VI) are between 93–95%, confirming the applicability and reliability of the developed analytical procedure.

In addition, the results for Cr (VI) content in waters obtained by the proposed analytical method were compared at a bit higher concentration level to the results obtained using a standard procedure based on the spectrophotometric method with 1,5-diphenylcarbazide (ISO 11083:1994). Natural ground waters from polluted aquifers in north Bulgaria were used for this comparison. The very good agreement observed between parallel results for more than 10 samples verifies the accuracy and versatility of the proposed approach for Cr(VI) quantification using hydrogel Cr(III)-IIM.

The experiments performed showed that the hydrogel membrane might be used for four adsorption/desorption cycles using 0.1 mol/L NH_4_-EDTA for elution (extraction efficiency above 95%). The extraction efficiency toward Cr(III) achieved by using hydrogel membranes from different batches showed very good repeatability, most probably due to the sustainability and robustness of the developed synthesis procedure.

### 2.7. Analytical Figures of Merit

Analytical figures of merit were defined after Cr(III) and Cr(VI) determination in five parallel samples. Detection and determination limits were calculated based on 3σ and 10σ criteria taking into account standard deviations of a blank sample (Cr measurement in 10 mL high-purity water passed through the whole developed analytical procedure. The results obtained are depicted in Table 5.

As seen from the results in Table 5, the analytical procedure developed is characterized by low determination limits and very good reproducibility. The most serious advantage is the possibility for direct determination of Cr(VI), avoiding any parallel determination and additional calculations.

A comparison of analytical figures of merit reported in the literature for Cr speciation procedures using different sorbent materials is presented in Table S2 [10,11,12,13,14,15,50]. As can be seen, the proposed in this work analytical method for the selective determination of Cr(III) and Cr(VI) ensures the lowest detection limits and allows the determination of environmentally relevant concentrations of Cr in surface/ground waters, even at background levels in unpolluted sites.

## 3. Conclusions

In this study, a novel hydrogel membrane, Cr(III)-imprinted poly(vinyl alcohol)/sodium alginate/AuNPs, was prepared by green synthesis and tested for Cr(III)/Cr(VI) separation. The formation of a double crosslinking interpenetrating polymer network and obtained good dispersion of gold nanoparticles in a polymer hydrogel matrix restricts the chain movements and thereby supports a mechanical strength of membrane produced and easy operation in sorption experiments. Optimization studies performed showed quantitative retention of Cr(III) at pH 6 and temperature 40 °C, while sorption of Cr(VI) is below 5%. The adsorption equilibrium for Cr(III) was attained within 20 h. The kinetics adsorption data for Cr(III) were well-fitted with a pseudo-first-order kinetic model, and the equilibrium data were best described by the Langmuir isotherm model. The maximum adsorption capacity of the Cr(III)-IIM for Cr(III) ions under the optimal condition was 1.75 mg/g. The successive adsorption–desorption experiment indicated that 0.1 mol/L NH_4_-EDTA solution could be effectively applied for Cr(III) elution from the Cr(III)-IIM, and the membrane can be used for additional three adsorption/desorption cycles.

A simple and sensitive analytical procedure was developed for the speciation of Cr in an aquatic environment using dispersive solid phase extraction of Cr(III) by Cr(III)-IIM membranes prior to selective Cr(VI) determination in the supernatants. The determination limit achieved for toxic species Cr(VI) fulfills the requirements for their monitoring in surface water bodies under the demand of the Water Frame Directive. The developed procedure avoids any additional calculations or parallel determinations for Cr(VI) quantification. In addition, if necessary, Cr(III) might be determined in the same sample with an even lower determination limit.

## 4. Materials and Methods

### 4.1. Materials, Reagents, and Instruments

High-purity water (Millipore Corp., Milford, MA, USA) was used to prepare all aqueous solutions. The working standard solutions were prepared daily by appropriately diluting the stock solutions of Cr(III) (Spex Certiprep 1000 mg/L in 2% HNO_3_) and Cr(VI) (Spex Certiprep 1000 mg/L in H_2_O).

Tetrachloroauric(III) acid (HAuCl_4_.3H_2_O, 99%, Panreac, Poland) and sodium tetrahydridoborate (NaBH_4_, GR for analysis, Merck, Germany) were used for AuNPs preparation. Sodium alginate (SA, low viscosity, Alfa Aesar, MA, USA), poly(vinyl alcohol) (PVA, relative molecular mass 72000, Sigma-Aldrich, St. Louis, MO, USA), and poly(ethylene glycol) (PEG, relative molecular mass 400, Sigma-Aldrich, St. Louis, MO, USA) were used to prepare the hydrogel Cr(III)-IIM and NIIM. Hydrochloric acid (Fisher Chemical™, Waltham, MA, USA) and ethylenediamine tetraacetic acid (EDTA, Sigma-Aldrich, St. Louis, MO, USA) were used for Cr desorption in the optimization experiments. After the dissolution of EDTA in NH_3_ solution (25%, Merck, Darmstadt, Germany), EDTA diammonium salt (NH_4_-EDTA) was prepared. The pH value of water samples was adjusted with NH_3_ solution or HNO_3_.

Absorption spectra of gold nanoparticles were recorded on a Thermo Scientific Evolution 300 UV–V spectrometer in the range 190–1100 nm, using quartz cuvette with 1 cm optical path. Quartz cuvette containing high-purity water served as a reference sample for background absorption. The morphology and sizes of gold nanoparticles were examined by a transmission electron microscope (TEM, JEOL JEM-2100 operating at 200 kV). The surface morphology of membranes was observed by scanning electron microscope (SEM, JEOL JSM-5510 operating at 10 kV). X-ray diffraction (XRD) patterns were registered on an X-ray powder diffractometer Siemens D500 equipped with the CuKα radiation (λ = 1.54 Å) in 2θ ranging from 10° to 90°.

The texture parameters were determined by nitrogen adsorption at temperature 77.4 K in NOVA 1200e (Quantachrome, Boynton Beach, FL, USA) instrument. The BET equation and the Gurvich rule (at a relative pressure close to 0.99) were used to calculate the specific surface area (*S*_BET_) T and the total pore volume (*V*_t_), respectively.

ATR-FTIR spectra were recorded by using Nicolet iS50 (Thermo Scientific, Waltham, MA, USA) Fourier Transform Infrared (FTIR) spectrophotometer with Attenuated Total Reflectance Attachment. In general, 32 scans and 4 cm^−1^ resolution were applied. The spectral data were processed with OMNIC Software (version 9.12.1002., (Thermo Fisher Scientific Inc., Waltham, MA, USA).

The concentrations of Cr were measured by Electrothermal atomic absorption spectrometry (Perkin Elmer Model AAnalyst 400, equipped with HGA 900 and AS 800 autosampler). Samples of effluate and eluate solutions (10–20 μL) were injected into pyrolytically coated graphite tubes using AS-800. Optimized temperature program consists of drying step at 120 °C, pretreatment step at 1100 °C, and atomization step at 2500 °C. Integrated absorbance signals (three replicates) were used for Cr quantification against external calibration.

The solutions’ pH was measured with a pH meter (Mettler Toledo; Seven Compact S220-K, Greifensee, Switzerland).

### 4.2. Synthesis of SA-AuNPs

The aqueous dispersions of sodium alginate stabilized gold nanoparticles were prepared by chemical reduction method based on the reduction of Au(III) (8 mL 0.001 mol/L HAuCl_4_) using strong reductant sodium tetrahydridoborate (24 mL 0.002 mol/L NaBH_4_) and alginate ions (1.5 mL 1% SA) as a non-toxic capping agent. The reduction was carried out in ice bath under magnetic stirring, and at the end of reaction sodium alginate solution was added for steric stabilization of gold nanoparticles by different functional groups, such as −COOH and −OH. The synthesis process is schematized in Figure S7. The noble metal nanoparticle dispersion was stored in dark bottles at room temperature. The wine-red dispersion of SA-AuNPs was stable for several months under storage conditions.

### 4.3. Preparation of Cr(III)-IIM and NIIM

The preparation of hydrogel Cr(III)-IIM includes using blends of poly(vinyl alcohol) and sodium alginate as film-forming materials, poly(ethylene glycol) as porogen agent, gold nanoparticles (SA-AuNPs) as cross-linking and mechanically stabilizing component, and Cr(III) ions as template species. In the typical procedure, aqueous solutions of SA (1% *w/v*) and PVA (2% *w/v*) were prepared in high-purity water with stirring at 85–90 °C for 90 min; then the hot solutions were filtered. Prepared mixture from PVA solution (30 mL) and PEG (115 mg) was poured into SA solution (30 mL) and stirred well for 30 min. This was followed by the addition drop by drop of Cr(III) solution (3 mL, 1000 mg/L), pH adjusting up to 5–6 by NaOH (2 mol/L). The resulting mixture was stirred vigorously for 60 min. In the next step, the pre-synthesized SA-AuNPs aqueous dispersion (80 mL) was added into the above polymer hydrogel matrix solution and stirred vigorously for 60 min. Then the solution was cast on plastic Petri dishes in portions of 7.5 mL and dried in hot air oven at 70 °C for 12 h. In order to remove the porogen PEG, the dried hydrogel membranes were immersed in high-purity water for one day. Then, chromium was extracted from the produced membranes by elution with 0.2 mol/L NH_4_-EDTA solution until the Cr concentration in the eluate solution was below the LOQ as measured by ETAAS. Similarly, in the absence of matrix ions, non-imprinted membranes (called NIIMs) were prepared. The whole imprinting process is schematized in Figure 1.

### 4.4. Static Adsorption/Desorption Experiments

The model solutions for static adsorption experiments were prepared by addition of 50 μg Cr(III) to 10 mL high-purity water. a The pH value between 4–9 was adjusted by HNO_3_ or NH_3_ solution. Cr(III)-IIM or NIIM was immersed in this solution and stirred with an electric shaker for 20 h at temperature of 40 °C. The membrane was removed and remaining solution (effluate) was analyzed by ETAAS. The membrane was treated twice with high-purity water, and Cr(III) was eluted with 0.1 mol/L NH_4_-EDTA solution. Chromium content was measured in the eluate by ETAAS.

The degree of sorption (*D*_S_, %) and degree of elution (*D*_E_, %) of Cr(III) ions were calculated by the following equations:

(8) $$D_{S}=\frac{A_{i}\;–A_{\mathrm{eff}}}{A_{i}}\times 100\;$$

(9) $$D_{E}=\frac{A_{\mathrm{el}}\;}{A_{i}–A_{\mathrm{eff}}}\times 100\;$$

where *A*_i_ (µg) is the initial amount of Cr(III) in contact with the membrane; *A*_eff_ (µg) is the amount of Cr(III) in the effluate solution after Cr(III)-IIM extraction; *A*_el_ (µg) is the amount of Cr(III) in the eluate.

### 4.5. Isotherm and Kinetic Studies

The following procedure was used for determination of the adsorption capacities of the hydrogel Cr(III)-IIM and NIIM: 10 mL solutions (pH 6) with various concentrations of Cr(III) ions (from 5 to 35 mg/L) were added to the tested membrane and shaken for 20 h at temperature 40 ± 1 °C. The Cr concentrations were measured in the effluate solutions by ETAAS under optimized instrumental parameters. All the experiments were performed in triplicate, and the average value was used to calculate the maximum adsorption capacity of Cr(III)-IIM and NIIM (*Q*_max,exp_) using the following expression:

(10) $$Q_{\max,\exp}=\frac{\left(C_{0}–C_{e}\right).V\;}{m}\;\;$$

where *Q*_max, exp_ (mg/g) is the mass of Cr(III) ions adsorbed per unit mass of the membrane; *V* (L)—solution volume; *m* (g)—mass of the membrane; *C*_0_ and *C*_e_ (mg/L)—initial and equilibrium concentrations of Cr(III) ions in the solution, respectively.

The sorption kinetics of Cr(III) was investigated using one Cr(III)-IIM in contact with 10 mL 5 mg/L Cr(III) standard solution at pH 6, placed in 15 mL centrifuge tubes on an electrical shaker at 150 rpm at 40 ± 1 °C. The sorption time was varied in the range of 1–24 h, and the residual Cr content in the effluate solutions was determined by ETAAS. Each experiment was repeated in triplicate. The amount of Cr(III) adsorbed at time *t*, *q*_t_ (mg/g), was calculated from Equation (11) by the difference between the initial chromium concentration in the solution (*C*_i_, mg/L) at *t* = 0 and the residual chromium concentration at *t* adsorption time (*C*_t_, mg/L):

(11) $$q_{t}=\frac{{(C}_{i}-C_{t}).V\;}{m}$$

### 4.6. Interference Studies on the Selective Separation of Cr(III) and Cr(VI)

A standard solution containing 50 μg Cr(III) or Cr (VI) was added separately to each one of the 10 mL model solutions, containing 5% NaCl, 400 mg/L SO_4_^2−^, 400 mg/L PO_4_^3−^, 100 mg/L Fe(III), Cu(II), or Zn(II) at pH 6. The hydrogel Cr(III)-IIMs were immersed in these solutions and stirred with an electric shaker for 20 h at temperature of 40 ± 1 °C. The membrane is removed, and remaining solution is analyzed by ETAAS. Chromium(III) content was quantified by ETAAS after membrane elution with 0.1 mol/L NH_4_-EDTA.

### 4.7. Analytical Procedure

A sample of surface water 20 mL was filtered through 45 µm membrane filter, and Cr(III)-IIM was immersed in this solution and stirred with an electric shaker for 20 h at temperature of 40 ± 1 °C. The supernatant solution is removed, and Cr(VI) is measured in this solution by ETAAS. In the case of very low concentrations of Cr (III), it might be eluted and also determined by ETAAS. The whole procedure could be performed during sampling—filtered sample is added to polypropylene vessel with inserted membrane. Supernatant after sorption is analyzed for Cr(VI) later in the laboratory.

## Figures and Tables

**Table 1:** Degree of elution *D*_E_ (%) of Cr(III) from Cr(III)-IIM using different eluents.

Eluent	*c*, mol/L	*D*_E_, %
HCl (V = 10 mL)	0.1	67.3 ± 3
	0.5	80.6 ± 3
	1.0	AuNPs dissolution
NH_4_-EDTA (V = 10 mL)	0.05	68.7 ± 4
	0.1	>99
	0.2	>99
NH_4_-EDTA (V = 5 mL)	0.1	75.6 ± 3
NH_4_-EDTA (V = 10 mL)	0.1	>99
NH_4_-EDTA (V = 20 mL)	0.1	>99

**Table 2:** Experimental adsorption capacity values and Langmuir and Freundlich isotherm parameters obtained by linear fitting for the Cr(III)-IIM and NIIM at temperature of 40 °C.

Polymer Hydrogel Membrane	*Q*_max,exp_ mg/g	Langmuir Isotherm Model				Freundlich Isotherm Model		
		*Q*_max,calc_ mg/g	*b* L/mg	*R* ^2^	*R*_L_	*k*_F_	*n*	*R* ^2^
Cr(III)-IIM	1.75	1.74	3.52	0.9997	0.01–0.05	1.15	3.47	0.8956
NIIM	1.23	1.25	0.32	0.9993	0.08–0.38	11.47	2. 38	0.9592

**Table 3:** Fitted kinetic parameters of pseudo-first-order, pseudo-second-order, Elovich, and intra-particle diffusion models for adsorption of Cr(III) ions onto the Cr(III)-IIM at concentration 5 mg/L, pH 6, temperature 40 °C, and adsorbent dose (one membrane) 0.140 g.

Model	Parameters	Values
Pseudo-first-order model	*k*_1_ (1/h)	0.07529
	*q*_e_*,*_calc_ * (mg/g)	0.4496
	*R* ^2^	0.9694
Pseudo-second-order model	*k*_2_ (g/(mg∙h))	0.07234
	*q*_e,calc_ * (mg/g)	0.6974
	*R* ^2^	0.9626
Elovich equation	*α* (mg/(g∙h))	0.09449
	β (g/mg)	8.1739
	*R* ^2^	0.9331
Intra-particle diffusion model Region 1	*k*_i_ (mg/(g∙h^0.5^)	0.1034
	*C*_i_ (mg/g)	−0.09989
	*R* ^2^	0.9558
Intra-particle diffusion model Region 2	*k*_i_ (mg/(g∙h^0.5^)	0.03459
	*C*_i_ (mg/g)	0.1892
	*R* ^2^	0.8710

* *q*_e_*,*_ex**p**_ = 0.3521 mg/g.

**Table 4:** Determination of Cr(III) and Cr(VI) in different types of waters (three parallel determinations).

Sample	Cr(III), µg/L Mean ± SD	Cr(VI), µg/L Mean ± SD	Recovery for Cr(VI), %
River water	2.3 ± 0.2	<DL	
River water + 0.5 µg/L Cr(VI)	2.2 ± 0.2	0.49 ± 0.02	94 ± 2
Seawater	0.52 ± 0.04	<DL	
Seawater + 0.2 µg/L Cr(VI)	0.54 ± 0.04	0.21 ± 0.02	95 ± 4
Groundwater	1.3 ± 0.1	0.25± 0.02	
Groundwater + 0.4 µg/L Cr(VI)	1.2 ± 0.1	0.63 ± 0.03	93 ± 4

**Table 5:** Analytical figures of merit determined after five parallel determinations.

Species	Detection Limit, µg/L	Determination Limit, µg/L	RSD, % for the Range 0.05–50 µg/L
Cr(III)	0.001	0.003	7–11
Cr(VI)	0.01	0.03	4–6

## Data Availability

Not applicable.

## Supplementary Materials

The following supporting information can be downloaded at: https://www.mdpi.com/article/10.3390/gels8110757/s1, Figure S1: (a) EDX mapping images and (b) EDX spectrum of Cr(III)-IIM after adsorption of Cr(III); Figure S2: FTIR spectra of Cr(III)-IIM and NIIM; Figure S3: Schematic presentation of the interactions between SA and Cr(III) ions; Figure S4: XRD patterns of (a) PVA/PEG/SA polymer membrane, NIIM, Cr(III)-IIM, SA-AuNPs (layer on glass slide); (b) Cr(III)-IIM, SA-AuNPs (layer on glass slide); Figure S5: Langmuir (a) and Freundlich (b) isotherms for adsorption of Cr(III) on the Cr(III)-IIM and NIIM; Figure S6: Adsorption kinetics of Cr(III) ions onto the Cr(III)-IIM at concentration 5 mg/L, pH 6, temperature 40 °C, and adsorbent dose (one membrane) 0.140 g: (a) pseudo-first order; (b) pseudo-second order; (c) Elovich, and (d) intra-particle diffusion model; Figure S7: Schematic representation of SA-AuNPs synthesis process; Table S1: Interference studies on the degree of sorption and the selectivity of Cr(III)-IIM hydrogel in the presence of different cations and anions in model solutions. (three parallel determinations); Table S2: Comparison of analytical figures of merit of analytical procedures using different sorbent materials for Cr(III)/Cr(VI) speciation.
