# Supplementary material for: Cr(III) Ion-Imprinted Hydrogel Membrane for Chromium Speciation Analysis in Water Samples
Source: Gels. 2022 Nov 21;8(11):757. doi: 10.3390/gels8110757 (PMC9689422; doi:10.3390/gels8110757)
Supplement: Supplementary file 1 [file gels-08-00757-s001.zip › gels-2029548-supplementary.pdf]

gels

MDPI

Supplementary Materials

Cr(III) Ion-Imprinted Hydrogel Membrane for Chromium Speciation Analysis in Water Samples

Ivanka Dakova *, Penka Vasileva and Irina Karadjova

Faculty of Chemistry and Pharmacy, University of Sofia "St. Kliment Ohridski", 1, J. Bourchier Blvd., 1164 Sofia, Bulgaria
* Correspondence: i.dakova@chem.uni-sofia.bg

Figures

Section: 2.2. *Characterization of SA-AuNPs and Cr(III)-IIM*

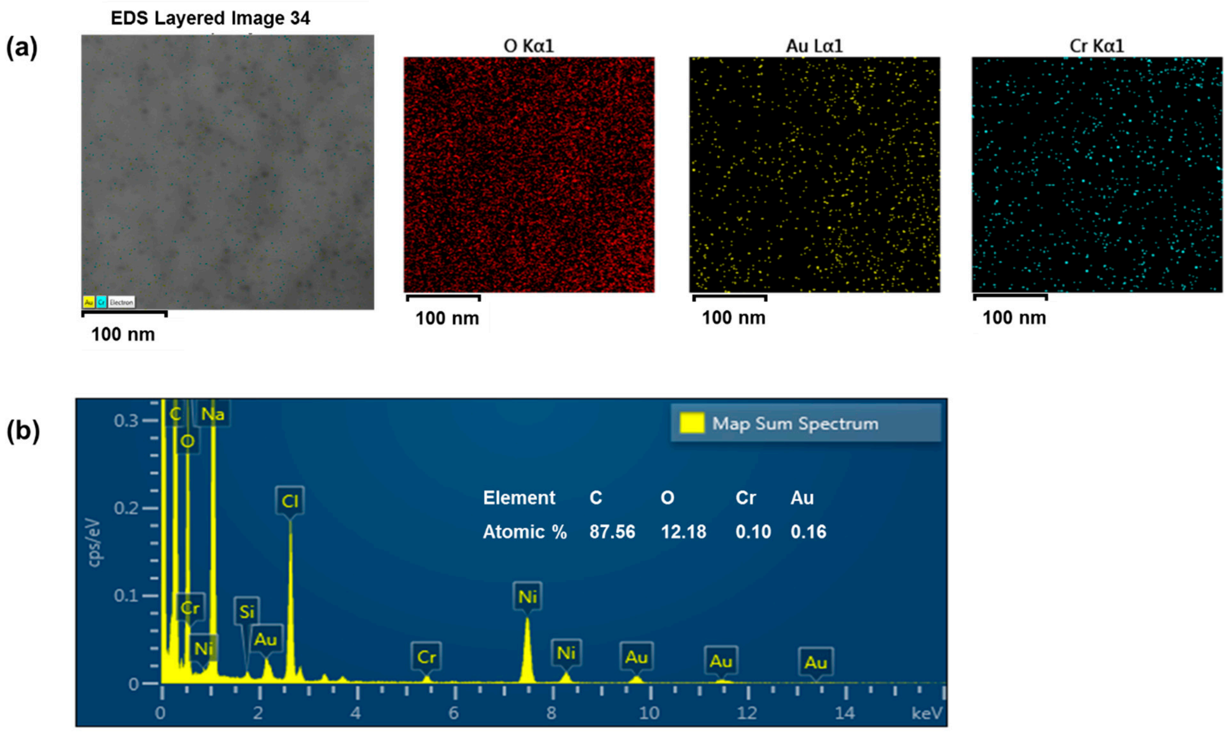

Figure S1. (**a**) EDX mapping images and (**b**) EDX spectrum of Cr(III)-IIM after adsorption of Cr(III).

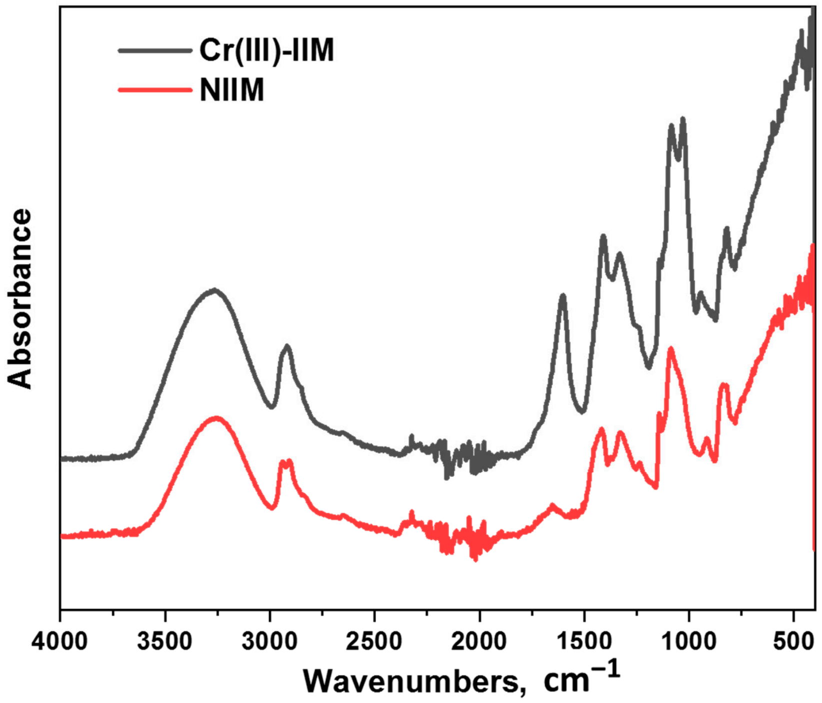

Figure S2. FTIR spectra of Cr(III)-IIM and NIIM.

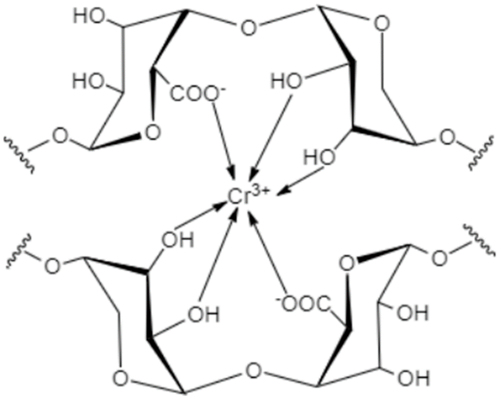

Figure S3. Schematic presentation of the interactions between SA and Cr(III) ions.

(a) (b)

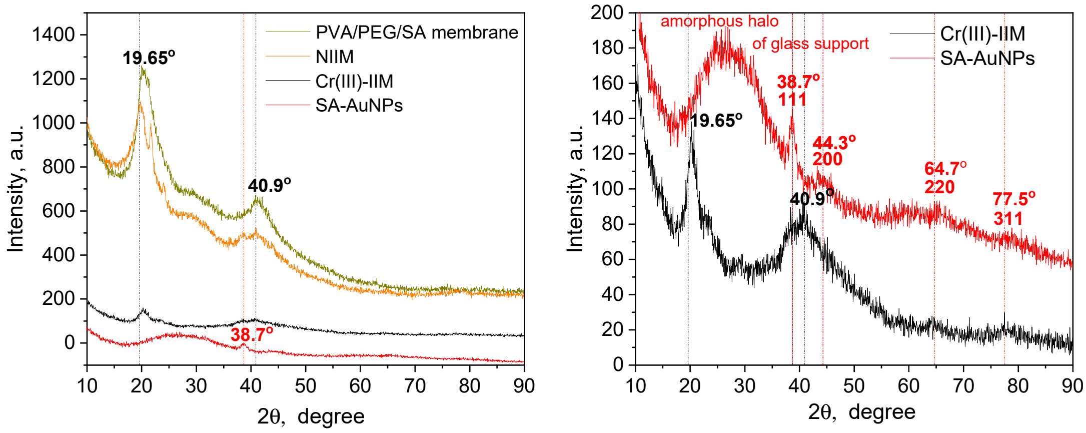

Figure S4. XRD patterns of (a) PVA/PEG/SA polymer membrane, NIIM, Cr(III)-IIM, SA-AuNPs (layer on glass slide); (b) Cr(III)-IIM, SA-AuNPs (layer on glass slide).

Section: *2.4.1. Adsorption isotherm models*

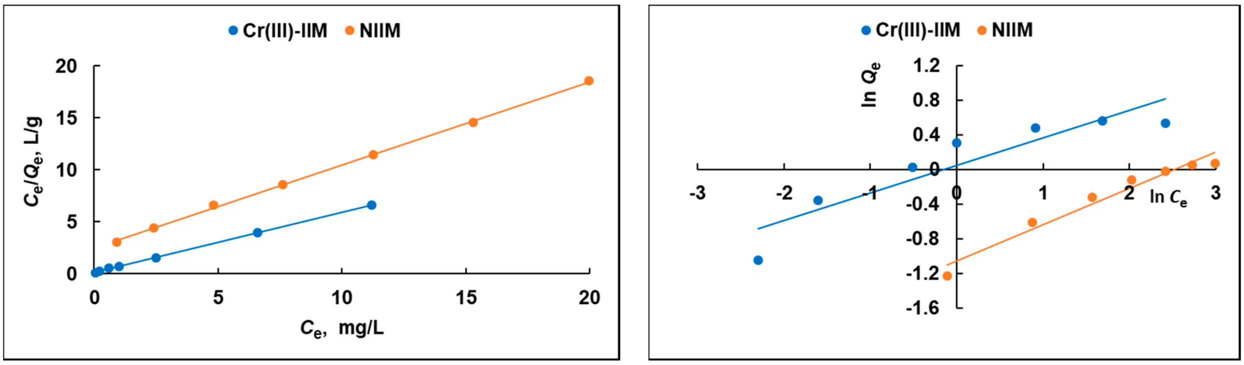

(a) (b)

Figure S5. Langmuir (a) and Freundlich (b) isotherms for adsorption of Cr(III) on the Cr(III)-IIM and NIIM.

Section: *2.4.2. Modelling of Cr(III) sorption kinetics*

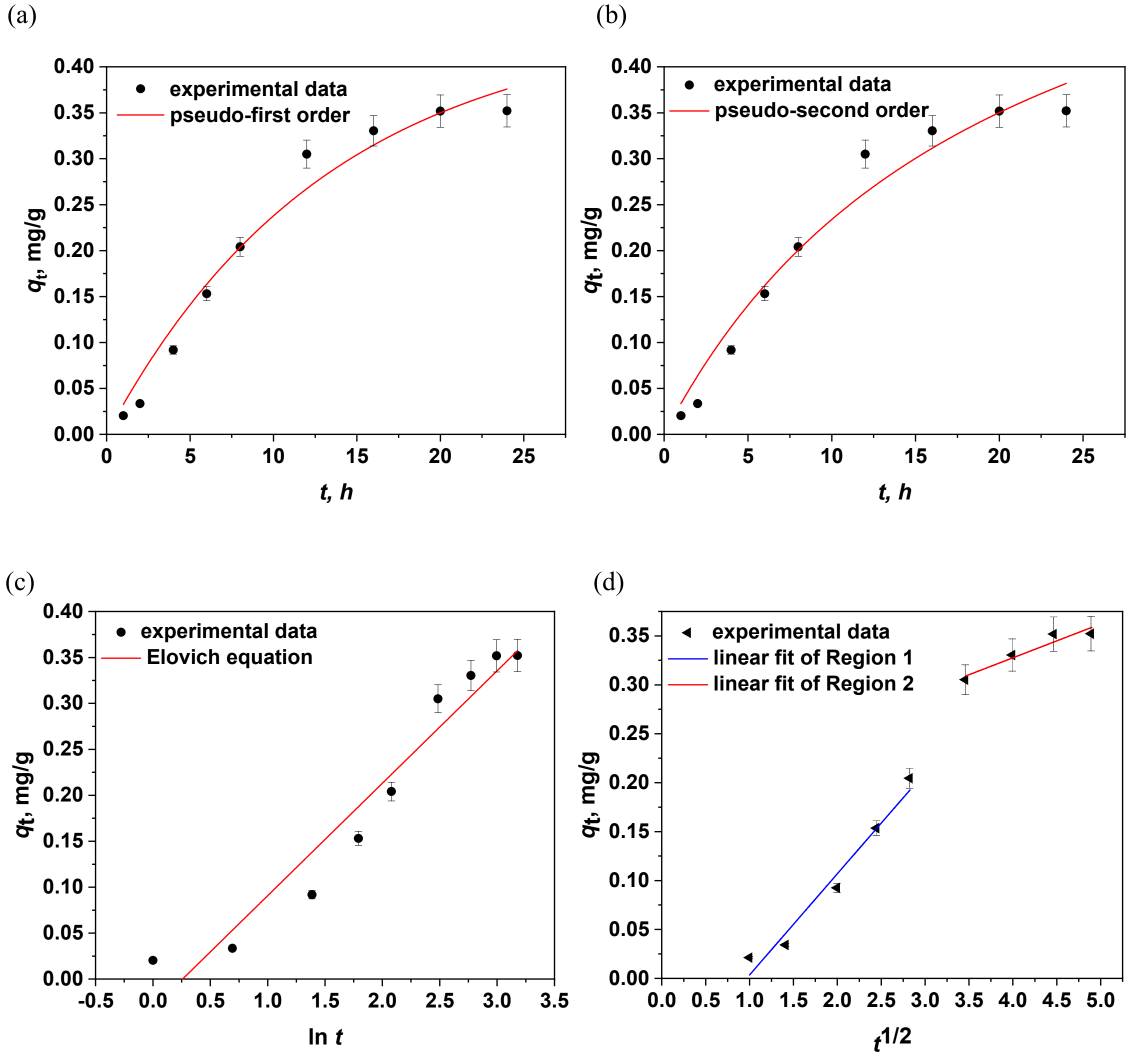

Figure S6. Adsorption kinetics of Cr(III) ions onto the Cr(III)-IIM at concentration 5 mg/L, pH 6, temperature 40 °C, and adsorbent dose (one membrane) 0.140 g: (a) pseudo-first order; (b) pseudo-second order; (c) Elovich, and (d) intra-particle diffusion model.

Section: *4.2. Synthesis of SA-AuNPs*

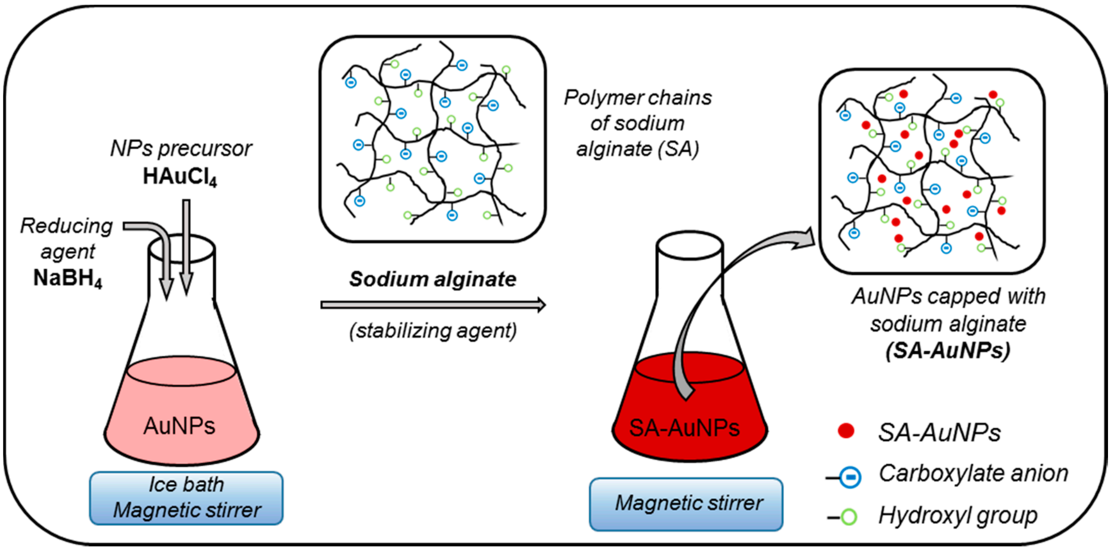

Figure S7. Schematic representation of SA-AuNPs synthesis process.

Tables

Section: *2.6. Analytical applications*

Table S1. Interference studies on the degree of sorption and the selectivity of Cr(III)-IIM hydrogel in the presence of different cations and anions in model solutions. (three parallel determinations);.

Interferent (concentration)	Degree of sorption, %	
	Cr(III)	Cr(VI)
NaCl (5%)	95 ± 2	<1
SO_4^{2-} (400 mg/L)	95 ± 3	<1
PO_4^{3-} (400 mg/L)	96 ± 3	<5
Fe(III) (100 mg/L)	97 ± 3	<5
Cu(II) (100 mg/L)	97 ± 2	<5
Zn(II) (100 mg/L)	97 ± 3	<5

Section: *2.7. Analytical figures of merit*

Table S2. Comparison of analytical figures of merit of analytical procedures using different sorbent materials for Cr(III)/Cr(VI) speciation.

Sorbent material	Sample	Instrumental measurement	Chemical species	LOD (μg/L)	RSD, % (at conc.)	Refs
Cr(III)-IIP based on Cr(III)-ethacryloylamido-histidine / EGDMA	water samples	FAAS and UV-vis	Cr(III), Total Cr	1.2	2.6	10
Cr(III) surface ion-imprinted silica gel	natural water samples	ICP-AES	Cr(III), Total Cr	0.11	1.2 (10)	11
Cr(III) mesoporous surface imprinted polymer adsorbent	plating and leather wastewater	ICP-AES and UV-vis	Cr(III), Total Cr	0.53	<2.1 (1000)	12
Cr(III)-IIP based on Cr(III)–pyrrolidinedithiocarbamate complex / acrylamide /EGDMA	tap and river water, municipal sewage	ETAAS	Cr(III), Cr(VI)	0.018	5.8 (5)	13
Cr(III)-IIP based on Cr(III)-8-hydroxyl-quinoline complex / styrene / divinylbenzene	CRM of waste water, municipal sewage samples	FAAS	Cr(III), Total Cr	2.18	3 (2000)	14
Cr(III)-imprinted 3-(2-aminoethylamino) propyltrimethoxysilane functionalized silica gel sorbent	environmental water samples	ICP-MS	Cr(III), Cr(VI)	0.004<br>0.008	4.44 (0.5)<br>4.41 (0.5)	15
Chitosan film loaded AgNPs	Water	ICP-MS	Cr(III), Cr(VI)	0.02<br>0.02	3–5 (0.05–5)	50
Cr(III) ion-imprinted hydrogel membrane	water samples	ETAAS	Cr(III),<br>Cr(VI)	0.001<br>0.01	7–11 (0.05–50)<br>4–6 (0.05–50)	This work
